# When the Single Matters more than the Group (II): Addressing the Problem of High False Positive Rates in Single Case Voxel Based Morphometry Using Non-parametric Statistics

**DOI:** 10.3389/fnins.2016.00006

**Published:** 2016-01-25

**Authors:** Cristina Scarpazza, Thomas E. Nichols, Donato Seramondi, Camille Maumet, Giuseppe Sartori, Andrea Mechelli

**Affiliations:** ^1^Department of Psychosis Studies, Institute of Psychiatry, Psychology and Neuroscience, King's College LondonLondon, UK; ^2^Department of Statistics, University of WarwickCoventry, UK; ^3^Warwick Manufacturing Group, University of WarwickCoventry, UK; ^4^Department of Human and Social Sciences, University of BergamoBergamo, Italy; ^5^Department of Psychology, University of PaduaPadova, Italy

**Keywords:** neuroimaging, magnetic resonance imaging, voxel based morphometry, single case study, non-parametric statistics, false positives

## Abstract

In recent years, an increasing number of studies have used Voxel Based Morphometry (VBM) to compare a single patient with a psychiatric or neurological condition of interest against a group of healthy controls. However, the validity of this approach critically relies on the assumption that the single patient is drawn from a hypothetical population with a normal distribution and variance equal to that of the control group. In a previous investigation, we demonstrated that family-wise false positive error rate (i.e., the proportion of statistical comparisons yielding at least one false positive) in single case VBM are much higher than expected (Scarpazza et al., 2013). Here, we examine whether the use of *non-parametric* statistics, which does not rely on the assumptions of normal distribution and equal variance, would enable the investigation of single subjects with good control of false positive risk. We empirically estimated false positive rates (FPRs) in single case non-parametric VBM, by performing 400 statistical comparisons between a single disease-free individual and a group of 100 disease-free controls. The impact of smoothing (4, 8, and 12 mm) and type of pre-processing (Modulated, Unmodulated) was also examined, as these factors have been found to influence FPRs in previous investigations using parametric statistics. The 400 statistical comparisons were repeated using two independent, freely available data sets in order to maximize the generalizability of the results. We found that the family-wise error rate was 5% for increases and 3.6% for decreases in one data set; and 5.6% for increases and 6.3% for decreases in the other data set (5% nominal). Further, these results were not dependent on the level of smoothing and modulation. Therefore, the present study provides empirical evidence that single case VBM studies with *non-parametric* statistics are not susceptible to high false positive rates. The critical implication of this finding is that VBM *can* be used to characterize neuroanatomical alterations in individual subjects as long as non-parametric statistics are employed.

## Introduction

The development of structural neuroimaging has allowed the *in vivo* investigation of the human brain. Over the past two decades, hundreds of studies have shed light on the neuroanatomical correlates of psychiatric (Honea et al., 2005; Fusar-Poli et al., 2011; Selvaraj et al., 2012) and neurological (Whitwell and Jack, 2005; Ferreira et al., 2011; Li et al., 2012) disorders. The vast majority of these studies were performed using Voxel Based Morphometry (VBM), a whole brain technique for characterizing regional volume and tissue concentration differences from structural magnetic resonance imaging (MRI) scans (Ashburner and Friston, 2000, 2001; Good et al., 2001; Mechelli et al., 2005). A typical VBM study compares a group of patients with a group of healthy controls, and tests for neuroanatomical differences between the two using group-level statistics. The results of these studies, however, have had limited translational impact in everyday clinical practice (Fusar-Poli et al., 2009; Ioannidis, 2011; Borgwardt et al., 2012), where a clinician needs to make inferences at the level of the individual patient. In recent years, an increasing number of research groups have attempted to overcome this by performing single case studies in which an individual patient is compared against a group of healthy controls (please see Scarpazza et al., 2013 for a summary of existing studies using single case VBM).

The interpretation of the results of parametric single case VBM studies, however, is problematic due to a number of methodological issues (Scarpazza et al., 2013). In particular, the use of two-sample *t*-tests requires the data to be sampled from normally distributed populations; therefore, under the null hypothesis, the validity of any single case VBM study relies on the assumption that the patient's value reflected a draw from a hypothetical normally-distributed population with variance equal to that of the control group population (see for Muhlau et al., 2009 for review). This issue was evaluated by Salmond et al. (2002), who examined false-positive rates in single case VBM as a function of the degree of smoothness applied to the data. The authors reported that the number of false positives was inversely related to the degree of smoothing and therefore suggested that VBM single case analysis could be performed as long as an appropriate smoothing kernel was applied (Salmond et al., 2002). In a subsequent investigation, Viviani et al. (2007a) examined the impact of non-normality on FPRs in the context of single case VBM studies. Using both simulated and empirical data, the authors reported that smoothing was only partially effective in compensating for the impact of deviation from normality (Viviani et al., 2007a). More recently, we empirically estimated the likelihood of detecting significant differences in gray matter volume (GMV) in individuals free from neurological or psychiatric diagnosis using two large, independent data sets (Scarpazza et al., 2013). We found that the chance of detecting a significant difference in a disease-free individual was much higher than expected; for instance, using a standard voxel-wise threshold of *p* < 0.05 (FWE corrected) and an extent threshold of 10 voxels, the likelihood of a single subject showing at least one significant difference was as high as 93.5% for increases and 71% for decreases. Consistent with earlier findings (Salmond et al., 2002), we also found that the chance of detecting significant differences was inversely related to the degree of smoothing applied to the data. Finally we found that FPRs were higher when examining tissue concentration using unmodulated data than when characterizing tissue volume using modulated data. We concluded that, when comparing a single neurological or psychiatric patient against a group of controls with VBM, the chance of detecting a significant difference not related to the disorder under investigation is much higher than expected. Interpretation of the results of single case studies should therefore be very cautious, particularly in the case of significant differences in temporal and frontal lobes where FPRs appear to be highest (Scarpazza et al., 2013).

One reason why VBM may *not* be a suitable analytical technique for making statistical inferences at the level of the individual patient is that it relies on parametric statistics which assume that the data are normally distributed or approximately normal by the Central Limit Theorem (CLT; Salmond et al., 2002). The CLT states that, for a sufficiently large number of identically distributed observations, the distribution of means will be approximately normally distributed (DeGroot et al., 1976). When this assumption is met, the parametric statistics used in VBM can be estimated reliably; in contrast, when this assumption is not met, test procedures may be susceptible to Type I errors. In VBM studies comparing different groups, the test resembles a two-sample *t*-test (except for the covariates, like age, and gender) and the two-sample *t*-test is quite robust to violations of the equal variance and normality assumptions *as long as* the sample sizes are equal (Posten, 1984; Posten et al., 2007). Single case VBM, where an individual patient is compared against a control group, is the most extreme case of an unbalanced two-sample comparison, and thus makes both the equal variance and normality assumptions critical.

The aim of the present investigation is to examine whether the use of a *non-parametric* permutation test method would enable the investigation of single subjects without the higher-than expected FPRs observed with parametric statistics. Whereas a parametric test assumes certain distributional forms to allow computation of *p*-values, a permutation test makes weak assumptions and uses the data itself to create empirical distributions of test statistics and ultimately *p*-values. For the two-sample *t*-test, the assumption is only that all the subjects are exchangeable under the null hypothesis; this implies that each subject would have the same distribution were there no group difference. For a General Linear Model (e.g., a two-sample *t*-test with additional covariates), the same assumption of exchangeability must be made on the additive errors. See Nichols and Holmes (2001) for a gentle introduction to permutation methods for neuroimaging and Winkler et al. (2014) for a detailed study of permutation methods for the GLM. Because non-parametric statistics do not require the data to be normally distributed, they may provide a valid alternative to parametric statistics in the context of single case VBM. Here, we conduct similar evaluation to the one described in Scarpazza et al. (2013), with the main difference being that here we used statistical non-parametric mapping (SnPM) as opposed to standard statistical parametric mapping (SPM). We empirically estimated the chance of detecting false positive differences in single case *non-parametric* VBM, by performing 100 comparisons between a single disease-free individual and a group of 100 healthy controls. As in the previous investigation (Scarpazza et al., 2013), the impact of smoothing and modulation on FPRs was investigated, as these factors have been found to influence the results of previous studies (Salmond et al., 2002; Viviani et al., 2007a). All statistical analyses were repeated using two independent freely available data sets, in order to maximize the generalizability of the results.

We examined three hypotheses. Our first hypothesis was that, when *non-parametric* statistics are used to compare a single subject to a control group in the context of VBM, FPRs would be valid (i.e., a FPR of 5% is expected for *p* < 0.05 FWE-corrected). Our second hypothesis was that, in the context of *non-parametric* statistics, smoothing and modulation would not affect false positives rates. Our third hypothesis was that, when they exist, false positives would be randomly distributed across the brain rather than being preferentially localized in specific regions (Scarpazza et al., 2015).

## Materials and methods

### Subjects

We used structural data from data from the 1000 functional connectomes data set (Biswal et al., 2010), available from the Neuroimaging Informatics Tools and Resources Clearinghouse (NITRC) at http://fcon_1000.projects.nitrc.org/fcpClassic/FcpTable.html. The Cambridge (Massachusetts, USA) and Beijing (China) data sets were chosen because of their large sample size (*n* = 198 each) and their similar age range (18–28). The Beijing data set is formed by 76 males and 122 females, mean age (standard deviation) = 21.1 ± 1.8 years. The Cambridge data set is formed by 75 males and 123 females, mean age (standard deviation) = 21 ± 2.3 years. A further reason for using these data sets was that all subjects were screened for having no history of neurological or psychiatric disorders.

### MRI data acquisition

A structural MRI scan was acquired from all subjects using a 3T MRI system. A T1-weighted sagittal three-dimensional magnetization-prepared rapid gradient echo (MPRAGE) sequence with full brain coverage was used. For the acquisition of the Cambridge data set, the following parameters were used: *TR* = 3 s, 144 slices, voxel resolution 1.2, 1.2, 1.2 mm^3^; matrix 192 × 192. For the acquisition of the Beijing data set, the following parameters were used: *TR* = 2 s, 128 slices, voxel resolution 1.0, 1.0, 1.3 mm^3^; matrix 181 × 175.

### Data analysis

#### Pre-processing

Images were checked for scanner artifacts and gross anatomical abnormalities; reoriented along the anterior–posterior commissure (AC–PC) line with the AC set as the origin of the spatial coordinates; segmented into gray matter (GM) and white matter (WM) using the segmentation procedure implemented in SPM8 (http://www.fil.ion.ucl.ac.uk/spm); and warped into a new study-specific reference space representing an average of all the subjects included in the analysis (Ashburner and Friston, 2009; Yassa and Stark, 2009), using a fast diffeomorphic image registration algorithm (DARTEL; Ashburner, 2007). As an initial step, two different templates (one for each data set) and the corresponding deformation fields, required to warp the data from each subject to the new reference space, were created using the GM partition (Ashburner and Friston, 2009). Each subject-specific deformation field was then used to warp the corresponding GM partition into the new reference space with the aim of maximizing accuracy and specificity (Yassa and Stark, 2009). Images were then affine transformed into Montreal Neurological Institute (MNI) space and smoothed with a 4, 8, and 12-mm full-width at half-maximum (FWHM) Gaussian kernel. The above procedure was followed twice to create both unmodulated and modulated images, which were analyzed separately. These two types of images provide different information: modulated data measure the absolute volume of gray matter, while unmodulated data measure the relative concentration of gray matter (Mechelli et al., 2005).

#### Group comparison

A single subject scan was compared with a control group made of 100 subjects. For each data set, the control groups were created from the total sample of 198 subjects using randomization as implemented in the following website: https://www.random.org/lists/. A sample size of 100 was chosen in order to allow enough unique permutations to accurately estimate the *p*-value (see below). For each data set we performed 400 comparisons using *non-parametric* statistics including the following: 100 comparisons between a single subject and 100 controls using modulated MRI images with a smoothing of 4 mm; 100 comparisons between a single subject and 100 controls using modulated MRI images with a smoothing of 8 mm; 100 comparisons between a single subject and 100 controls using modulated MRI images with a smoothing of 12 mm; and 100 comparisons between a single subject and 100 controls using unmodulated MRI images with a smoothing of 8 mm. The analyses on unmodulated data were performed on subjects with smoothing 8 mm only for consistency with our previous investigation (Scarpazza et al., 2013).

#### Statistical analysis using non-parametric statistics

The statistical analysis of MRI data using *non-parametric* statistics was performed using the Statistical Non-Parametric Mapping (SnPM 13.0.11) toolbox, available at http://warwick.ac.uk/snpm (Nichols and Holmes, 2001). This toolbox uses *non-parametric* permutation testing to identify significant increases or decreases in each subject relative to a control group. For each statistical comparison, the *p*-value was estimated using a total of 101 permutations based on a control group size of 100. Age and gender were entered into the design matrix as covariates of no interest to minimize any impact of these variables on the findings. To exclude voxels outside brain, we used a relative threshold mask to discard voxels whose intensity fell below the 20% of the mean image intensity. To identify regionally specific changes that were not confounded by global differences, we used the proportional scaling option. Statistical inferences were made voxel-wise using Family-wise Error (FWE) correction for multiple comparisons across the whole brain at *p* < 0.05. No extent threshold was used since the main aim of the current investigation was to quantify the number of false positive results irrespective of cluster size.

For each data source (Beijing and Cambridge) we recorded the count of family-wise errors, or false positives (out of 100), over the three smoothing kernels (4, 8, and 12 mm), two pre-processing types (Modulated, Unmodulated) and two directions (increases and decreases in a single subject compared to a control group).

In order to investigate whether smoothing and direction of the effect had a significant impact on the number of false positives in the context of modulated data, we fit a logistic regression model for counts from each data source, using the presence of a family-wise error in each comparison (yes or no) as dependent variable, and smoothing and direction as independent variables. For 8 mm smoothing both modulated and unmodulated data were available, and therefore we fit a further logistic regression model for each data source; here the dependent variable was the presence of a statistically significant difference in each comparison (yes or no), and the independent variables were modulation and direction (with only 8 mm smoothing available, smoothing and sample size were not modeled). Both logistic regression models were assessed with the Hosmer–Lemeshow goodness-of-fit test, where a significant *p*-value indicates lack-of-fit.

#### Brain areas individuation

From the SnPM output, i.e., the list of MNI coordinates of the areas showing significant increases or decreases, we derived the corresponding areas using the Automated Anatomical Labeling (AAL) atlas as implemented in PickAtlas software (http://fmri.wfubmc.edu/software/PickAtlas).

## Results

### Number of comparisons yielding significant differences

Table 1 shows the empirical family-wise error rate, i.e., the percentage of statistical comparisons yielding at least one false positive, for each smoothing kernel and each data set. Over all settings, the error rate never exceeded 8%, and all were well within the 95% Monte Carlo confidence interval for 100 realizations (0.7–9.3%).

**Table 1:** **Number of significant differences**.

		**4 mm**		**8 mm**		**12 mm**	
		**Increase**	**Decrease**	**Increase**	**Decrease**	**Increase**	**Decrease**
NP-modulated	Beijing	6 (8)	8 (11)	4 (6)	6 (9)	5 (5)	5 (8)
	Cambridge	7 (15)	4 (4)	5 (12)	2 (2)	5 (7)	5 (6)
NP-unmodulated	Beijing	–	–	2 (2)	6 (6)	–	–
	Cambridge	–	–	3 (4)	4 (4)	–	–
P-modulated	Beijing			48 (79)	31 (41)		
	Cambridge			51 (70)	27 (44)		

*Percentage of statistical comparisons yielding at least one false positive (at p < 0.05 FWE corrected) across different statistics (P, parametric; NP, non-parametric), smoothing kernels (4, 8, 12 mm) and for both modulated and unmodulated data. The number in brackets refers to the total number of clusters detected across statistical comparisons*.

### Impact of smoothing and direction

The Hosmer–Lemeshow test for both regressions was non-significant (*p* = 0.999 and 0.821, for Beijing and Cambridge data sets, respectively), consistent with a null hypothesis of good model fit. The impact of smoothing on the family-wise error was not significant, in either the Beijing (*p* = 0.328) or the Cambridge (*p* = 0.673) data set. Direction had a significant impact on family-wise error was significant in the Beijing data (*p* = 0.024) but not in the Cambridge data set (*p* = 0.127).

### Impact of modulation and direction

The Hosmer–Lemeshow test for both regressions was not significant (*p* = 0.795 and 0.547, for Beijing and Cambridge data sets, respectively), consistent with a null hypothesis of good model fit. The impact of modulation on the family-wise error was not significant, in either the Beijing (*p* = 0.629) or the Cambridge (*p* = 0.991) data set. Direction did not have a significant impact on family-wise error in either the Beijing (*p* = 0.156) or the Cambridge (*p* = 0.588) data set.

### Likelihood of detecting local maxima in a specific region

**Figure 1:**
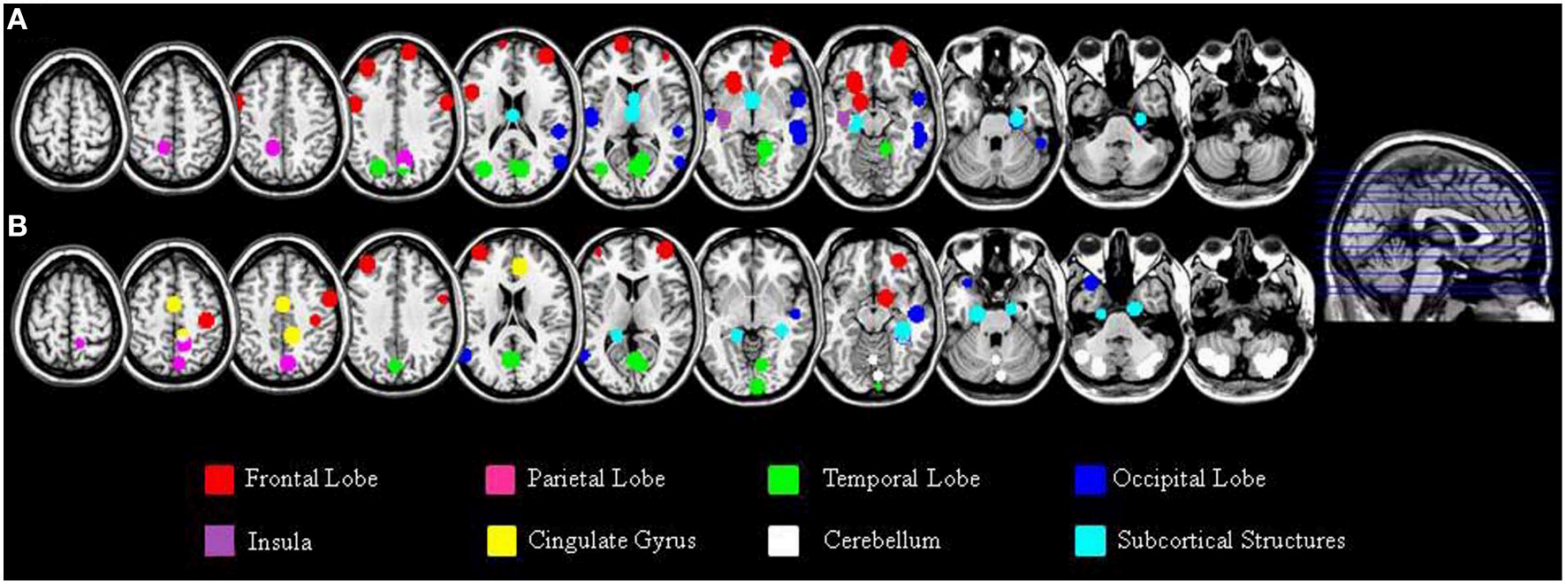
**Localization of the false positives, based on the peak coordinates, in the Beijing (A) and Cambridge (B) data sets across all statistical analyses with modulated images**. This image was created using the peak coordinates; a 10 mm radius was chosen for display purposes in order to make each peak clearly visible.

In addition to the empirical family-wise error rate, we also examined the location of the false positives. Given the small number of false positives, we report this information across the three smoothing kernels (4, 8, and 12 mm) and across directions (increases, decreases) using modulated data. A total of 47 false positives were detected in the Beijing data set and a total of 46 false positives were detected in the Cambridge data set. The distribution of these false positives across the brain, based on the peak coordinates, is summarized in Table 2 and represented graphically in Figure 1. In addition, the exact region in which each peak was located is reported in the Supplementary Material Table S1.

**Table 2:** **The table reported the volume in mm^3^ of each cerebral region**.

	**Volume mm^3^**	**Volume percentage (%)**	**Beijing (*n* = 46 clusters)**		**Cambridge (*n* = 47 clusters)**	
			**Raw number**	**Percentage (%)**	**Raw number**	**Percentage (%)**
Frontal lobe	562.6	35.5	15	32.6	7	14.8
Parietal Lobe	214.8	13.51	2	4.3	2	4.2
Temporal Lobe	258.7	16.29	10	21.7	4	8.5
Occipital Lobe	170.6	10.73	13	28.2	6	12.7
Insula	29	1.83	1	2.1	0	–
Cingulate	61.2	3.85	0	–	3	6.3
Subcortical structures	89.7	5.62	6	13	5	10.6
Cerebellum	196.9	12.38	0	–	19	40.4

*The percentage has been calculated on a total of 1583 mm^3^ of total intracranial volume. Absolute number and proportion of statistically significant differences in different cortical and subcortical areas were reported for Beijing and Cambridge data sets, separately*.

A large proportion of the false positives were localized in the cortex (40 out of 47 corresponding to 86.8% of the total number in the Beijing data set; 19 out of 46 corresponding to 44.8% of the total number, in the Cambridge data set) whereas only a small fraction were detected in subcortical regions (6 false positives corresponding to 13% in the Beijing data set; 5 false positives corresponding to 10.6% in the Cambridge data set). In addition, the false positives did not appear to be equally distributed across the cortex; rather, they were mainly located in the frontal lobe (15 false positives out of 46 corresponding to 32.6% in the Beijing data set; 7 false positives out of 47 corresponding to 14.8% in the Cambridge data set) and in the occipital lobe (13 false positives out of 46 corresponding to 28.2% in the Beijing data set; 6 false positives out of 47 corresponding to 12.7% in the Cambridge data set) compared to the other lobes (temporal: 10/46, 21.7%, and 4/47, 8.5% in the Beijing and Cambridge data sets respectively; parietal: 2/46, 4.3% and 2/47, 4.2% in the Beijing and Cambridge data sets respectively). We considered the possibility that the preferential localization of false positives in the frontal lobe might reflect its larger size (35.5% of the total brain volume) relative to other cortical lobes (13.5% for the parietal lobe; 16.2% for the temporal lobe, and 10.7% for the occipital lobe). In order to explore this possibility, we used Spearman's correlation to estimate the association between the volume (mm^3^ and percentage) of the regions in Table 2 and the number of false positives in these regions, for the two data sets separately. This association was not significant, either in the Beijing (*R* = 0.58, *p* = 0.12) or the Cambridge (*R* = 0.47, *p* = 0.23) data set. Therefore, the idea that the preferential localization of false positives in the frontal lobe might reflect its larger size was not supported.

### Comparison with parametric statistics

In the present investigation, a control group of 100 healthy controls was required to allow enough unique permutations to accurately estimate the *p*-value. In contrast, in our previous investigation of FPRs in single case VBM with *parametric* statistics (Scarpazza et al., 2013), we used a control group of 16 healthy volunteers. Therefore, in order to compare FPRs for *non-parametric* and *parametric* statistics without the confound of different sample sizes, we performed 100 comparisons between a single disease-free individual and a group of 100 healthy controls using *parametric* statistics for each of the two data sets. Critically, the very same control groups were used in the two sets of analyses, allowing us to interpret any difference in FPRs as a result of the type of statistics. The statistical comparisons using *parametric* statistics were performed using Statistical Parametric Mapping (SPM8) software, available at http://www.fil.ion.ucl.ac.uk/spm/. For the comparisons testing significant increases in a single subject relative to a control group, we found an error rate of 48 and 51% for the Beijing and Cambridge data sets respectively; in contrast, for the comparisons testing significant decreases in a single subject relative to a control group, error rates for the Beijing and Cambridge data sets were about 31 and 27% respectively (see Table 1).

## Discussion

Although VBM was initially developed to detect subtle differences between groups (Ashburner and Friston, 2000, 2001; Good et al., 2001; Mechelli et al., 2005), this analytical technique is increasingly being used to examine neuroanatomical abnormalities in individual subjects (Scarpazza et al., 2013). Our previous investigation showed that VBM is *not* a reliable technique for investigating single cases due to high susceptibility to false positive findings (Scarpazza et al., 2013). We suggested that this was explained by VBM's reliance on parametric statistics, which require the patient data to respect the assumption of normal distribution and to reflect the mean value of a hypothetical patient population with a variance equal to that of the control group. In the present study we aimed to investigate whether *non-parametric* VBM, which does not rely on parametric statistics, allows the investigation of individual subjects without high susceptibility to false positive findings. This was achieved by empirically estimating the likelihood of detecting significant differences when comparing a single subject against a control group comprising of 100 subjects.

We tested three related hypothesis. Firstly and most importantly we hypothesized that, when *non-parametric* statistics are used, FPRs would be as expected theoretically (e.g., around 5%) and therefore much lower than the ones detected using parametric statistics (Scarpazza et al., 2013). Secondly we hypothesized that FPRs would not vary as a function of smoothing and modulation. Thirdly, we hypothesized that false positives would be randomly distributed across the brain rather than being preferentially localized in specific regions.

We found that, across the three smoothing kernels investigated, the average number of statistical comparisons yielding at least one false positive was 5% for increases and 3.6% for decreases in the Beijing data set; and 5.6% for increases and 6.3% for decreases in the Cambridge data set. These FPRs are considerably lower than the very high FPRs observed with parametric statistics, which reached approximately 50% for increases and 30% for decreases. Thus, consistent with our first hypothesis, single case VBM with *non-parametric* statistics is not susceptible to the high FPRs observed in the context of single case VBM with parametric statistics. The critical implication of this finding is that VBM *can* be used to investigate individual subjects as long as appropriate (i.e., *non-parametric*) statistics are employed.

In line with our second hypothesis, we found that, in the context of *non-parametric* statistics, the FPRs were not affected by the degree of smoothing applied to the data. This aspect of our results is consistent with our previous investigation comparing balanced groups using parametric statistics, which also found a very small number of false positive findings (Scarpazza et al., 2015). However, it is inconsistent with previous studies comparing a single subject against a group using parametric statistics that reported high FPRs (Salmond et al., 2002; Viviani et al., 2007a; Scarpazza et al., 2013). This can be explained by the fact that, in the context of parametric statistics, the degree of smoothing affects the normality of the data, which in turn determines the validity of the test. On the other hand, in the context of non-parametric statistics, the test is not affected by the normality of the data (and therefore by the degree of smoothing). In line with our second hypothesis, we also found that FPRs did not differ for unmodulated and modulated data. This is in accordance with our previous study comparing balanced groups using parametric statistics (Scarpazza et al., 2015) and in contrast with our previous investigation comparing a single subject against a group using parametric statistics (Scarpazza et al., 2013). The fact that smoothing and modulation did not have a significant effect on the results is encouraging since, if VBM using non-parametric statistics is a valid approach in the context of single case studies, then the same FPRs should be expected regardless the smoothing and modulation applied to the data. However, we cannot exclude the possibility that the very small number of false positive findings in the present investigation may have reduced the statistical power to detect the impact of these factors compared to our previous study (Scarpazza et al., 2013).

In addition, we found that a large proportion of false positive findings were expressed in the cortex in both data sets, which is likely to reflect its larger size compared with subcortical structures. However, in the Beijing data set the percentage of false positives located in the cortex was 86.8%, while in the Cambridge data set it was only 44.8%. This discrepancy between data sets can be explained by the fact that, in the Cambridge data set, 19 out of 47 false positives (40.4%) were located in the cerebellum; however, as mentioned in the Results, 17 out these 19 false positives came from a single statistical comparison which was a clear outlier (see Supplementary Table S1 for details). We also found that the majority of false positives within the cortex were located in frontal lobe as opposed to the parietal, occipital or temporal regions. We examined the possibility that the preferential localization of false positives in the frontal lobe might reflect its larger size relative to other cortical lobes (Semendeferi et al., 1997). This possibility, however, was not supported by correlation analyses investigating the relationship between regional volume and number of false positives (see Section Likelihood of Detecting Local Maxima in a Specific Region of the Results). There are at least three additional explanations for the non-random spatial distribution of the false positives across the brain: firstly, there is a higher degree of neuroanatomical variability in the frontal lobe than in other cortical lobes (Casey et al., 2000; Carreiras et al., 2009; Fleming et al., 2010); secondly, the spatial distribution of overthreshold peaks is thought to be associated with the local degree of smoothness (Taylor and Worsley, 2007) as indexed by the Resolution Element (RESEL) map (Worsley et al., 1992); thirdly, overthreshold peaks are more likely to occur in areas where skeweness or kurtosis is more marked (Viviani et al., 2007a,b). When a single case is compared against a comparison group, as in the current investigation, the combined effect of the above systematic sources of non-homogeneity might lead to the occurrence of false positives in some regions more than others. On the other hand, we note that the impact of neuroanatomical variability, RESEL maps, skewness and kurtosis would be expected to decrease with a higher degree of smoothing, which was not the case in the present work. Also this aspect of our results should be considered with caution, since it is based on a relatives small number of false positives.

Taken collectively, these results have important implications for studies using single case VBM to characterize neuroanatomical alterations in individual patients relative to a control group. A major challenge for these studies, which in the past have always been conducted using parametric statistics, is the high rates of false positives which results from the violation of the assumption of normality (Scarpazza et al., 2013). Here we have shown that this challenge can be overcome with the use of *non-parametric* statistics, which do not require the data to have a normal distribution. A significant strength of the present study is that all statistical analyses were repeated using two independent data sets composing individuals from distinct ethnic groups (i.e., Caucasian and Chinese). Overall the results were highly consistent across the two data sets, providing support to the idea that the current results can be generalized to other research centers. The present study has a number of limitations. Firstly, the statistical comparisons carried out within each data set were not completely independent. This is because, since the control groups comprised of 100 subjects randomly selected from a data set of 198 subjects, the same subjects would be present in different control groups. A second important limitation is that, although the permutation test computes valid rejection thresholds irrespective of whether or not the data are normally distributed, it does not compensate for the unequal occurrence of false positives due to the unequal spatial occurrence of non-normality. A third limitation is that our interpretation of the results is based on the assumption that all subjects were free from neurological or psychiatric disorders. Although the subjects are free from any diagnosis, we cannot exclude the possibility that some of them might have experienced subclinical symptoms that were reflected in neuroanaomical alterations. Fourth, a control group of 100 individuals was required to allow enough permutations to accurately compute the *p*-value. In this context the number of possible permutations N_P_ is equal to the control group size plus 1, and permutation *p*-values are multiples of 1/N_P_. Permutation *p*-values are valid (control false positive risk), but with only 16 control subjects, as in our previous investigation, the smallest possible *p*-value is 1/17 = 0.0588. It could be argued that the requirement for such large control group makes the use of non-parametric statistics impractical in a clinical setting, where it might be difficult to acquire neuroanatomical scans from 100 healthy controls.

In conclusion, the present study provides empirical evidence that single case VBM with *non-parametric* statistics is not susceptible to high FPRs. The critical implication of this finding is that VBM *can* be used to characterize of neuroanatomical alterations in individual subjects as long as non-parametric statistics are employed. Although there are still significant theoretical and practical challenges for the translational implementation of single case VBM in neurology and psychiatry, the present findings suggest that VBM could become a potentially valuable clinical tool. Having established the validity of single case non-parametric VBM, future studies could examine the sensitivity of this analytical approach to neuroanatomical alterations using data from neurological and psychiatric populations.

## Author contributions

CS, GS, and AM design the work; CS, DS, TN, CM analyzed the data; all the authors contributed to the interpretation of the data; CS and AM provided an initial draft of the manuscript; TN, DS, CM, and SG provided insightful and critical revision of the manuscript.

## Funding

This research was supported by a grant (ID99859) from the Medical Research Council (MRC) to AM.

### Conflict of interest statement

The authors declare that the research was conducted in the absence of any commercial or financial relationships that could be construed as a potential conflict of interest.
