# Supplementary material for: When the Single Matters more than the Group (II): Addressing the Problem of High False Positive Rates in Single Case Voxel Based Morphometry Using Non-parametric Statistics
Source: Front Neurosci. 2016 Jan 25;10:6. doi: 10.3389/fnins.2016.00006 (PMC4724722; doi:10.3389/fnins.2016.00006)
Supplement: Supplementary file 1 [file Table1.docx]

**SUPPLEMENTARY MATERIAL:**

**When the single matters more than the group (II): addressing the problem of high false positive rates in single case Voxel Based Morphometry using non-parametric statistics.**

Scarpazza C, Nichols T, Seramondi D, Maumet C, Sartori G, Mechelli A

**Table S1**. Location of each detected false positive for each analysis performed using modulated images. Localization was performed using the Automated Anatomical Labeling (AAL) atlas as implemented in PickAtlas software (<http://fmri.wfubmc.edu/software/PickAtlas>). Subject refer to the code of the single subjects compared with a control group of 100 randomly selected subjects.

|  | **Smoothing 4mm** | | | **Smoothing 8 mm** | | | **Smoothing 12 mm** | | |
| --- | --- | --- | --- | --- | --- | --- | --- | --- | --- |
| **B**  **E**  **I**  **J**  **I**  **N**  **G** | **Subject** | **Increases** | **Decreases** | **Subject** | **Increases** | **Decreases** | **Subject** | **Increases** | **Decreases** |
|  | Subj98617 | Parahippocampus_R |  | Subj00440 | Frontal_Sup_Orb_R |  | Subj00440 | Frontal_Mid_Orb_R |  |
|  | Subj29590 | Putamen_R |  |  | Frontal_Mid_Orb_R |  | Subj29590 | Thalamus |  |
|  | Subj08251 |  | Lingual_R | Subj29590 | Caudatus_R |  | Subj37602 | Frontal_Inf_Orb_L |  |
|  | Subj46541 |  | Heschl_L |  | Thalamus |  | Subj08251 | Insula_L |  |
|  |  |  | Heschl_L | Subj37602 | Frontal_Inf_Orb_L |  | Subj74587 |  | Cuneus_R |
|  | Subj35776 | Olfactory_L |  | Subj74587 |  | Cuneus_R |  |  | Frontal_Sup_R |
|  | Subj19642 | Temporal_Mid_R |  | Subj75878 |  | Occipital_Sup_L |  |  | Precuneus_R |
|  | Subj74587 |  | Cuneus_R | Subj81062 | Temporal_Mid_R | Temporal_Mid_R | Subj66889 |  | Precentral_L |
|  | Subj66889 |  | Precentral_L | Subj35309 |  | Calcarine_R | Subj92544 | Frontal_Inf_Tri_L |  |
|  | Subj88947 |  | Precentral_R |  |  | Calcarine_R | Subj88947 |  | Occipital_Mid_L |
|  | Subj10973 |  | Temporal_Sup_R |  |  | Calcarine_L |  |  | Occipital_Mid_L |
|  | Subj35309 |  | Lingual_R | Subj20127 |  | Hippocampus_L | Subj35309 |  | Calcarine_R |
|  |  |  | Frontal_Sup_Medial-L |  |  | Precuneus_L | Subj56757 |  | Temporal_Inf_R |
|  |  |  | Calcarine_R | Subj56757 |  | Temporal_Inf_R |  |  |  |
|  | Subj77440 | Frontal_Mid_R |  |  |  |  |  |  |  |
|  |  | Frontal_Mid_Orb_R |  |  |  |  |  |  |  |
|  |  | Frontal_Mid_L |  |  |  |  |  |  |  |
|  | Subj91399 | Fusiform_R |  |  |  |  |  |  |  |
|  | Subj76377 |  | Temporal_Pole_Sup_R |  |  |  |  |  |  |
| **C**  **A**  **M**  **B**  **R**  **I**  **D**  **G**  **E** | Subj51512 |  | Frontal_Sup_L | Subj68101 | Precentral_R |  | Subj68101 |  | Precuneus_R |
|  | Subj69315 | Temporal_Mid_R |  | Subj51512 |  | Parahippocampal_R | Subj51512 |  | Frontal_Mid_R |
|  | Subj53296 | Cuneus_L |  | Subj53296 | Cuneus_L |  |  |  | Frontal_Sup_R |
|  | Subj00294 |  | Cingulum_Ant_L |  | Calcarine_R |  | Subj53296 | Vermis_6 |  |
|  | Subj43304 | Hippocampus_L |  |  | Cuneus_L |  |  | Lingual_R |  |
|  | Subj69287 | Frontal_Mid_Orb_R |  | Subj02591 | Cerebelum_Crus2_R |  | Subj01361 |  | Cingulum_Mid_R |
|  | Subj02591 | Cerebelum_Crus2_R |  |  | Cerebelum_8_R |  | Subj02953 |  | Precuneus_R |
|  |  | Cerebelum_Crus2_R |  |  | Cerebelum_Crus2_R |  | Subj02591 | Cerebelum_Crus2_R | Fusiform_R |
|  |  | Cerebelum_8_R |  |  | Cerebelum_Crus2_L |  |  | Cerebelum_7b_L |  |
|  |  | Cerebelum_8_R |  |  | Cerebelum_8_L |  | Subj84064 | Temporal_Pole_Mid_L |  |
|  |  | Cerebelum_Crus2_L |  |  | Cerebelum_Crus2_L |  | Subj47498 | Parahippocampal_L |  |
|  |  | Cerebelum_8_L |  | Subj09633 | Cingulum_Mid_L |  | Subj34586 | Cerebelum_Crus1_R |  |
|  |  | Cerebelum_Crus2_R |  | Subj47498 | Cingulum_Mid_L |  |  |  |  |
|  |  | Cerebelum_Crus1_R |  | Subj34586 |  | Calcarine_L |  |  |  |
|  |  | Cerebelum_Crus1_R |  |  |  |  |  |  |  |
|  | Subj07413 |  | Frontal_Mid_R |  |  |  |  |  |  |
|  | Subj63661 | Temporal_Mid_L |  |  |  |  |  |  |  |
|  | Subj50454 | Frontal_Mid_R |  |  |  |  |  |  |  |
|  | Subj99330 |  | Hippocampus_R |  |  |  |  |  |  |
